# Effects of testosterone and exercise training on bone microstructure of rats

**DOI:** 10.14202/vetworld.2022.627-633

**Published:** 2022-03-22

**Authors:** Catarina Jota-Baptista, Ana I. Faustino-Rocha, Margarida Fardilha, Rita Ferreira, Paula A. Oliveira, Marta Regueiro-Purriños, José A. Rodriguez-Altonaga, José M. Gonzalo-Orden, Mário Ginja

**Affiliations:** 1 Department of Veterinary Medicine, Surgery and Anatomy, Institute of Biomedicine (IBIOMED), University of León, Léon, Spain; 2 Centre for the Research and Technology of Agro-Environmental and Biological Sciences (CITAB), Inov4Agro, Vila Real, Portugal; 3 Department of Zootechnics, School of Sciences and Technology, Évora, Portugal; 4 Comprehensive Health Research Center (CHRC), Évora, Portugal; 5 iBIMED, Department of Medical Sciences, University of Aveiro (UA), Aveiro, Portugal; 6 LAQV-Associated Laboratory for Green Chemistry (REQUIMTE), Department of Chemistry, UA, Aveiro, Portugal; 7 Department of Veterinary Sciences, University of Trás-os-Montes and Alto Douro, Vila Real, Portugal; 8 Animal and Veterinary Research Center (CECAV), Vila Real, Portugal

**Keywords:** bone, exercise training, hypogonadism, micro-computed tomography, osteoporosis, testosterone

## Abstract

**Background and Aim:**

Male hypogonadism results from failure to produce physiological levels of testosterone. Testosterone in men is essential in masculine development, sperm production, and adult man’s health. Osteoporosis is one of the consequences of hypogonadism. Regular physical exercise and exogenous testosterone administration are frequently used to prevent or treat this condition. This study aimed to understand the effects of lifelong exercise training and testosterone levels (isolated and together) in the main bone structure parameters.

**Materials and Methods:**

A total of 24 rats were used and randomly divided into four groups: Control group (CG; n=6), exercised group (EG, n=6), testosterone group (TG, n=6), and testosterone EG (TEG, n=6). A micro-computed tomography equipment was used to evaluate 15 bone parameters.

**Results:**

Both factors (exercise training and testosterone) seem to improve the bone resistance and microstructure, although in different bone characteristics. Testosterone influenced trabecular structure parameters, namely, connectivity density, trabecular number, and trabecular space. The exercise promoted alterations in bone structure as well, although, in most cases, in different bone structure parameters as bone mineral density and medullar mineral density.

**Conclusion:**

Overall, exercise and testosterone therapy seems to have a synergistic contribution to the general bone structure and resistance. Further studies are warranted, comparing different individual factors, as gender, lifestyle, or testosterone protocols, to constantly improve the medical management of hypogonadism (and osteoporosis).

## Introduction

According to the Endocrine Society Clinical Practice Guideline, male hypogonadism is “a clinical syndrome that results from failure of the testes to produce physiological levels of testosterone due to disruption of one or more levels of the hypothalamic-pituitary-testicular axis” [1]. Men hypogonadism may come from primary hypogonadism (testicular dysfunction) or central hypogonadism (hypothalamic-pituitary dysfunction). Testosterone in men is essential in masculine development, sperm production, and adult man’s health. The most frequent consequences include a decrease in hair growth, a decrease in muscle mass, gynecomastia (development of breast tissue), and osteopenia, a remarkable reduction of bone mineral density (BMD). BMD is defined as the volumetric density of calcium hydroxyapatite in tissue [2]. Osteoporosis happens when the patient has a reduced bone mass with micro-architectural deterioration (as “pores”). These conditions are dependent on the reabsorption and formation of bone tissues. When bone reabsorption exceeds bone formation, bone mass decreases, which leads to osteopenia and osteoporosis [3]. The most frequent clinical consequences include chronic pain and spontaneous fractures. More than 20% of older men (over 60) have age-related hypogonadism [4].

The use of long-term testosterone to prevent hypogonadism clinical consequences, namely, the decrease of BMD, is now broadly used [4]. Testosterone directly affects the androgen receptor on osteocytes and osteoblasts, increasing trabecular bone formation, and preventing reabsorption [5]. Besides its positive impact on bone structure, this therapy also improves erectile function, libido, and muscle strength. Even though the clinical benefits are totally recognizable, potential risks must be analyzed in each clinical case. Those potential adverse effects involve prostate disorders (including benign prostatic hyperplasia), erythrocytosis, or some dermatologic lesions. Moreover, it can lead to reduced spermatogenesis due to the suppression of luteinizing hormone, being inappropriate to improve or maintain fertility. Nowadays, several testosterone formulations are available, and some additional formulations are currently under development, including injectable or transdermal formulations and subcutaneous pellets [6]. Regular physical activity is also important to improve bone density and preventing lesions associated with lower bone density [3]. Inactivity can cause bone loss and decrease BMD [7]. Mechanical tension and loading can successfully improve bone strength, being a crucial regulator of skeletal development. Many authors have studied and demonstrated the positive relationship between exercise training and bone density in older women [7,8] and men [9-11]. However, the results are often controversial and inconsistent. Some authors suggest that bones become less sensitive to mechanical loading after maturity (age 18-25 years) [12]. From a systematic review, others conclude that controlled exercise (with calcium supplementation) causes a modest reduction of bone resorption, resulting in only 1-2% gain in BMD*/*year in women [13]. Nevertheless, both International Osteoporosis Foundation and Royal Osteoporosis Society recommend exercise to patients to improve their bone density and prevent or treat osteoporosis and subsequent fractures [14]. In combination, exercise training and testosterone therapy are broadly recommended to prevent or treat osteoporosis and other consequences of hypogonadism in men, even when other clinical conditions are also present, as obesity or diabetes *mellitus* [15]. Computed tomography (CT) is a complementary diagnostic examination that involves taking X-ray images from many angles around body tissues, converting them into sectional slices, allowing a 3D perspective [16]. Micro-CT is designed for small objects due to its higher resolution. In this context, it allows a detailed analysis of the trabecular structure of small animals’ bones or biopsy specimens. Rather than only evaluate BMD, that is, broadly used in osteoporosis studies, micro-CT provides information on various parameters as cortical bone porosity, cortical volume (CV), medullar bone volume (BV), and the number of trabeculae. In other words, it turns the morphometric evaluation of the specimen into an histomorphometric study, giving a deeper perspective of the bone structure [17,18]. Therefore, the 3D representations can be used to create computer simulations of bone remodeling and it can also be used to dynamically evaluate a therapy response (e.g., testosterone) [19]. Micro-CT is progressively more used to study osteoporosis and other bone lesions in mice and rats, such as Src homology 2 domain–containing inositol polyphosphate 5′-phosphatase (SHIP)-knockout, parathyroid hormone-related protein heterozygous null, and mice with Zmpste24 deficiency [20].

This study aims to unveil the effects of lifelong exercise training and testosterone administration (isolated and together) in the main bone structure parameters, using micro-CT equipment in a prostate cancer rat model.

## Materials and Methods

### Ethical approval

All the experiments were approved by the Institutional Animals Ethics Committee and by Portuguese national authority (Direção Geral de Alimentação e Veterinária, approval number 021326).

### Study period and location

This study was conducted from September 2016 to October 2017 at Animal House, University of Trás-os-Montes and Alto Douro, Portugal.

### Animals

Twenty-four Wistar Unilever male (4 weeks old) rats were bought from Charles River (Écully, France). The animals were submitted to a period of acclimatization after their arrival to guarantee a proper adaptation to the animal facilities of the University of Trás-os-Montes and Alto Douro. Throughout the study, they were kept under controlled conditions of temperature (22±2ºC), humidity (50±10%), and light: dark cycle (12 h:12 h). Food (Mucedola 4RF21^®^, Milan, Italy) and water were provided *ad libitum*.

### Experimental protocol

Animals were randomly divided into four experimental groups with six animals each: Control group (CG), exercised group (EG), testosterone group (TG), and testosterone exercised group (TEG). Food and water consumption were recorded every week during the study, and individual body weight was registered once a month.

At 8 weeks of age, the animals from exercised groups (EG and TEG) started the exercise program in a treadmill (Treadmill Control LE 8710, Harvard Apparatus, USA), for 53 weeks (5 days*/*week). The exercise started with a week of habituation, during which the exercise program had a duration of 30 min*/*day. Then, it was increased to 60 min*/*day for the rest of the experiment. The treadmill’s speed was initially set to 70% of the maximal speed capacity of the animals. Every 6 weeks, the speed capacity was reevaluated to correct exercise intensity. To submit the sedentary groups (CG and TG) to similar stress, those rats were frequently placed on a stationary treadmill for a few minutes.

Animals from testosterone-treated groups (TG and TEG) were exposed to a protocol for prostate cancer induction. At 12 weeks old, these animals received a subcutaneous administration of the anti-androgenic drug flutamide (50 mg/kg; TCI Chemicals, Portland, OR, USA) for 21 consecutive days. Twenty-four hours after the last flutamide administration, testosterone propionate (TCI Chemicals, Portland, OR, USA) was dissolved in corn oil and then administered by subcutaneous injection to the animals at a dose of 100 mg/kg. Forty-eight hours later, they were intraperitoneally injected with the carcinogen agent *N*-methyl-*N*-nitrosourea (Isopac^®^, Sigma Chemical Co., Madrid, Spain), at a dose of 30 mg/kg. Two weeks later, testosterone implants were subcutaneously implanted in the interscapular region of animals until the end of the experimental protocol, which were previously anesthetized with ketamine (75 mg/kg, Imalgene^®^ 1000, Merial S.A.S., Lyon, France) and xylazine (10 mg/kg, Rompun^®^ 2%, Bayer Healthcare S.A., Kiel, Germany). The testosterone implants were made from silastic tubing (Dow Corning, from VWR Scientific or other reliable sources; ID 0.078 in; OD 0.125 in.) sealed with G.E. RTV-108 adhesive sealant. Briefly, the implants were filled with 3 cm tightly packed crystalline testosterone (Sigma Chemical Co., Madrid, Spain) with the aid of a small spatula and tamped with a clip previously sterilized in an autoclave to ensure that all tubes had the same amount of testosterone. The tubes were always kept upright, and the sealant was placed on the tube ends after the tubes were completely filled.

**Figure-1:**
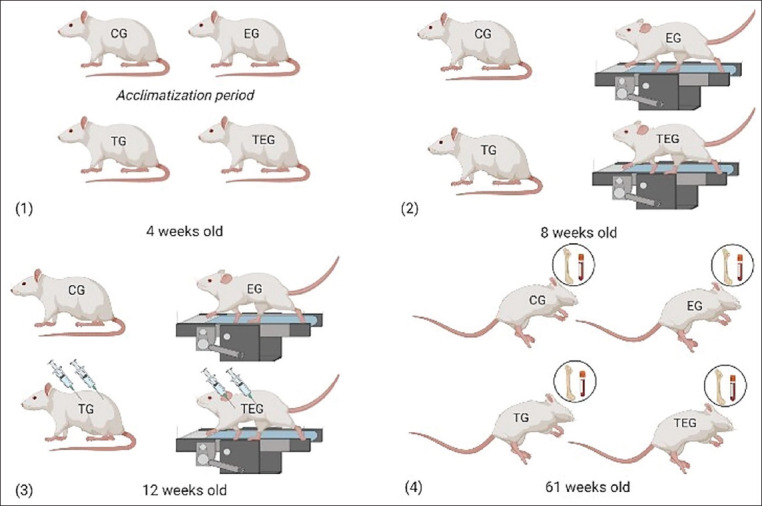
Experimental protocol performed in each one of the four groups: Control group (CG), exercised (EG), testosterone group (TS), and testosterone exercised group (TEG). (1) Quarantine and acclimatization period; (2) initiation of the exercise training; (3) initiation of protocol for testosterone treatment; and (4) animals’ sacrifice and samples’ collection.

At 61 weeks of age, all animals were sacrificed through an intraperitoneal injection of ketamine (75 mg/kg, Imalgene^®^ 1000, Merial S.A.S., Lyon, France) and xylazine (10 mg/kg, Rompun^®^ 2%, Bayer Healthcare S.A., Kiel, Germany) followed by exsanguination by cardiac puncture (10-12 mL of blood was collected from each animal). A complete necropsy was performed and the right femur of each animal was collected. The experimental protocol is illustrated in Figure-1.

### Blood analysis

Testosterone concentration was adequately measured by an ELISA Kit (582701; Cayman Chemical, MI, USA), according to the manufacturer’s instructions. The lower limit of detection of this assay is 5 pg/mL, the % CV is 4.4-19.1% (intra-assay) and 7.7-14.2% (interassay).

### Femur preparation and micro-CT examination

The right femurs were dissected and cleaned from all soft tissues. They were then placed in a sample cylindrical holder in 10% formalin solution for 48 h, washed in a phosphate-buffered saline solution and preserved in 70% ethanol. The cylindrical sample holder was placed in a vertical position with the femoral head down and was imaged separately at an isotropic resolution of 16.7 mm using a micro-CT device (SkyScan 1174v.2; Bruker micro-CT, Kontich, Belgium). The scanner parameters were set at 50 kV, 800 mA, 360 rotations around the vertical axis, and a rotation step of 0.3 using a 1 mm thick aluminum filter. Scanning was done in the femur diaphysis and distal metaphyseal region. The acquired projection images were reconstructed into cross-sectional slices in a total of more than 1500 images using GPUReconServer 1.7.0.4 software (SkyScan, Aartselaar, Belgium).

The microarchitectural characteristics of trabecular bone were evaluated in the metaphyseal region by selecting 200 transversal images nest to the distal growth plate, and cortical bone was evaluated by selecting 150 images in the diaphyseal region.

Eighteen parameters were used to evaluate our samples: Tissue volume (TV) in a region of interest (ROI) (cortical bone including pores) (TV ROI), BV in ROI (cortical bone without pores) (BV ROI), medullary volume in ROI (Md. V. ROI), percent BV (BV/TV), tissue surface (TS), bone surface (BS), intersection surface (iS), BS/volume ratio (BS/BV), BS density (BS/TV), CV, total porosity (Po(tot)), medullary BV (MBV), trabecular number (Tb.N), trabecular thickness (Tb.Th), trabecular separation (Tb.Sp), and connectivity density (Conn.D). The tissue mineral density (TMD) and BMD were also determined. BMD is the combined density of a specific section containing a mixture of bone and medullar tissue, while the TMD excludes this tissue [21,22].

### Statistical analysis

SPSS^®^ program version 27 (Statistical Package for the Social Sciences, Chicago, IL, USA) and Microsoft Office Exce^l®^ (Microsoft, Washington, USA) were used for statistical analysis. Results are presented as mean±standard deviation. Statistical differences among groups were assessed by one-way analysis of variance for independent samples, followed by Bonferroni *post hoc* tests. p<0.05 was considered statistically significant.

## Results

### Testosterone serum levels

For each group, the mean testosterone serum levels are presented in Table-1. The testosterone serum levels were significantly higher in testosterone-treated groups (TG and TEG), when compared with non-treated groups (CG and EG) (p<0.05). Differences were not found in the testosterone levels between non-treated groups and between treated groups (p>0.05).

**Table-1:** Testosterone serum levels in each experimental group (mean±standard deviation).

Group (n=6)	Testosterone (pg/mL)
CG	134.22±74.14^#^
EG	284.28±111.65^#^
TG	1813.72±953.69^$^
TEG	2654.47±57.47^$^

Values with different symbols were considered statistically different (p<0.05). CG=Control group, EG=Exercised group, TG=Testosterone group, TEG=Testosterone exercised group

### Micro-CT examination: Bone parameters

Bone parameters addressed by micro-CT in all experimental groups are presented in Table-2. Generally, the bone parameters were higher in testosterone-treated animals (TG and TEG) when compared with non-treated groups (CG and EG), especially considering trabecular bone structure. For instance, Tb.N and MBV were higher in TG and TEG, while Tb.Sp was lower, compared to CG and EG. In TEG, Po(tot) had a wide range of results (from 0.08 to 1.39, with a medium value of 0.73), while it was homogeneous and consistently lower in other groups.

**Table-2:** Bone parameters addressed by micro-computed tomography (micro-CT) in all experimental groups (mean±standard deviation).

Group (n=6)	CG	EG	TG	TEG
TV ROI (mm^3^)	27.71±2.75	29.52±2.75	29.90±0.92	29.02±2.09
BV ROI (mm^3^)	17.72±1.08	19.21±2.28	20.02±0.77	19.74±1.84
Md.V ROI (mm^3^)	9.99±0.87	10.31±0.60	9.88±0.67	9.28±0.55
BV/TV (%)	63.83±1.17^#^	65.00±1.90^#,$^	67.00±1.67^$^	67.83±2.14^$^
TS (mm^2^)	56.33±2.80	58.33±4.26	59.00±1.41	57.83±2.79
BS (mm^2^)	72.33±3.20	76.00±5.02	76.00±1.67	79.67±6.62
iS (mm^2^)	40.33±1.37	42.17±2.32	42.00±1.26	41.50±1.64
BS/BV (mm)	4.10±0.12	3.97±0.26	3.83±0.08	4.03±0.23
BS/TV (mm)	2.62±0.10^#,$^	2.57±0.08^#,$^	2.55±0.08^#^	2.73±0.15^$^
CV (mm^3^)	17.72±1.08	19.21±2.28	20.02±0.77	19.74±1.84
Po (tot) (%)	0.12±0.12^#^	0.11±0.05^#^	0.17±0.16^#^	0.73±0.48^$^
MBV (%)	16.27±1.30^#^	21.69±2.20^$^	19.21±2.11^#,$^	22.09±1.94^$^
Tb.N (per mm)	1.73±0.17^#^	2.44±0.17^$,&,*^	2.10±0.23^&^	2.55±0.23^*^
Tb.Th (micra)	94.25±3.76^#^	88.89±4.63^#,$^	91.67±2.94^#,$^	86.78±2.45^$^
Tb.Sp (micra)	500.04±41.88^#^	335.03±25.58^$^	396.88±55.95^$^	319.32±23.39^$^
Conn.Dn (*per* mm^3^)	28.96±8.21^#^	63.00±4.83^$,*^	47.85±2.74^$,*^	58.18±5.47^$,*^
BMD (g/cm^3^)	0.27±0.02^#^	0.35±0.03^$^	0.31±0.04^#,$^	0.36±0.04^$^
TMD (g/cm^3^)	1.26±0.02	1.26±0.02	1.26±0.01	1.27±0.01

TV ROI=Tissue volume in a region of interest (ROI) (cortical bone including pores); BV ROI=Bone volume in ROI (cortical bone without pores); Md.V ROI=Medullary volume in ROI; BV/TV=Percent bone volume; TS=Tissue surface; BS=Bone surface; iS=Intersection surface; BS/BV=Bone surface/volume ratio; BS/TV=Bone surface density; CV=Cortical volume; Po (tot)=Total porosity; MBV=Medullary bone volume; Tb.N=Trabecular number; Tb.Th=Trabecular thickness; Tb.Sp=Trabecular separation; Conn.Dn=Connectivity density; BMD=Bone mineral density; TMD=Tissue mineral density. For each parameter, values with different symbols were considered statistically different (p<0.05). CG=Control group, EG=Exercised group, TG=Testosterone group, TEG=Testosterone exercised group

The statistical analysis demonstrated differences among groups in nine (9/18) bone parameters: BV/TV, BS/TV, Po(tot), MBV, Tb.N, Tb.Th, Tb.Sp, Conn.Dn, and BMD (p<0.05). The bone characteristics of animals from EG were more similar to those from animals exposed to testosterone (TG and TEG) than with those from CG (p>0.05).

No differences were found in ROI volume parameters (TV ROI, BV ROI, and Md. V ROI) and surface parameters (TS, BS, and iS) among the four groups. In contrast with BMD, TMD showed no statistical differences among the four groups.

## Discussion

A total of 18 bone parameters were analyzed and compared with testosterone serum levels and exercise training, considering the different study groups. Results were compared with other animal or human studies to discuss and present informative and practical data on osteoporosis and hypogonadism clinical approaches. The bone changes evidenced in TG and TEG groups can be assumed due to exposure to high levels of testosterone, as statistically supported. Our study revealed consistently higher MBV, Tb.N, and BMD values in exercise groups, which is in agreement with the Canadian Multicentre Osteoporosis Study recommendations. This study clearly reinforces the role of exercise in increasing BMD and preventing osteoporosis, as previously reported in both men and women [23]. However, comparing EG and TEG, we had very similar results for BMD (0.35±0.03 and 0.36±0.04, respectively). One study compared the BMD of two elder men groups subjected to intensive lifestyle intervention (exercise program); one received testosterone therapy and the other received a placebo. No significant changes in BMD were observed between those groups [24], which may reinforce our result. However, some authors argue that exercise has a limited impact on BMD, increasing this value by 1-2%*/*year only [13].

On the other hand, testosterone is a recognized therapeutic option for hypogonadism clinical consequences, namely, reduced bone resistance, due to its physiological action on bone tissue [25]. A testosterone ester mixture every week for 3 months of 6 hypogonadal patients caused a small increase (7%) in BMD at the lumbar spine, according to photon absorptiometry [26]. Nevertheless, sublingual testosterone cyclodextrin (5 mg, 3 times/day) for 6 months in 34 hypogonadal men did not change the BMD of the hip or spine, measured by dual-energy X-ray absorptiometry [4,27].

As illustrated, different techniques of BMD measurements, selected bones, exercise programs, and testosterone protocols have been used in distinct studies, leading to different conclusions [4,25,26]. Therefore, more multifactor studies and bone parameters are needed to identify which type of exercise and testosterone protocol are better to improve bone resistance.

TMD does not reveal any changes in our study among the four groups, unlike BMD. As mentioned above, BMD is the combined density of the bone and bone marrow, which makes it suitable to evaluate the trabecular bone. TMD, in contrast, excludes the medullar region, which makes it an accurate way to evaluate cortical bone tissue [28]. This may suggest a more pronounced effect of exercise and testosterone in the trabecular bone, though many studies report improved densities in both cortical and trabecular regions [29,30].

A previous study studied the testosterone effects by comparing bone micro-CT analysis between normal and castrated males, which have lower levels of this hormone. In one experiment, orchidectomy caused a reduction in trabecular bone Conn.D (TConn.Dn) [31]. In another, castration significantly decreased TConn.Dn and Tb.N. These findings are consistent with ours, since these parameters also differed among our groups. Explicitly, TConn.Dn and Tb.N are higher in one or both T groups, compared to CG [32]. Moreover, Tb.Sp was significantly higher in CG. Therefore, this also supports the fact that testosterone levels strongly influence trabecular bone structure [2,33].

Unexpectedly, porosity (Po(tot)) was significantly higher in TEG, with a wide range of results (from 0.08 to 1.39%). Most studies reported a decrease of bone porosity in people receiving testosterone therapy to prevent bone fractures. In contrast, teriparatide therapy improves femur general strength but increases porosity, making it inappropriate for most types of osteoporosis [33,34]. Some authors refer that bone porosity and its evolution with age are different according to the region of the bone evaluated [35], which may explain our results.

Nevertheless, osteoporosis is a hormone-related problem not only in men but also in women. Estrogen deficiency predisposes to osteoporosis in postmenopausal women. A study shows significantly lower BV/TV and Tb.Th; and substantially higher Tb.N and Tb.Sp in ovariectomized rats compared to normal rats [36]. Although both estrogens and androgens stimulate bone formation, the signaling pathways involved are distinct and the risk of fracture is consistently higher in women. It would be very interesting to compare both sexes and bone effects of those deficiencies, using micro-CT, and to verify why osteoporosis should be managed with little differences in men and women [37,38].

According to our results, both factors of our study (exercise training and testosterone) seem to generally improve the bone resistance and microstructure (especially in trabecular bone), although in different bone characteristics and properties. This long-term study undoubtedly emphasizes the advantages of using both in combination for several comorbidities of hypogonadism [39].

## Conclusion

Animals from testosterone-treated groups presented changes in bone structure that can be related to a higher testosterone exposure. Lifelong exercise training promoted alterations in bone structure as well, although, in most cases, in different bone structure parameters. Thus, exercise and testosterone therapy seems to have a synergistic contribution to the general bone structure and resistance. Micro-CT represents a high-quality examination that can provide detailed information on bone histological changes, giving a complete picture of the bone dynamics, rather than analyzing only one parameter, as BMD. Furthermore, by comparing different individual factors, such as gender, lifestyle, or testosterone protocols, it would be possible to improve the management of hypogonadism (and osteoporosis) constantly.

## Authors’ Contributions

CJ: Prepared the manuscript. PAO: Designed the experimental protocol. PAO, MG, and AIF: Carried out and monitored the animal experiments. MR, JAR, and JMG: Carried out the bone analysis. PAO, MG, MF, RF, and AIF: Revised the manuscript. All authors read and approved the final manuscript.
